# Lesbian, gay, bisexual, and transgender clinical competence of physiotherapy students in Israel

**DOI:** 10.1186/s12909-024-05679-6

**Published:** 2024-07-05

**Authors:** Michal Elboim-Gabyzon, Roei Klein

**Affiliations:** 1 Department of Physical Therapy, Faculty of Social Welfare and Health Sciences , University of Haifa, 188 Hushi Abba Boulevard, Haifa, 3498837 Israel; 2 Department of Physical Therapy, Recanati School for Community Health Professions, Faculty of Health Sciences, Ben-Gurion University of the Negev, Beer Sheva, Israel

**Keywords:** Clinical competence, Physiotherapy students, Lesbian, Gay, Bisexual, Transgender development of clinical skills scale

## Abstract

**Background:**

Clinical competence encompasses attitudes, skills, and knowledge regarding diverse client groups. Appropriate clinical competence requires an understanding of the cultural context in which healthcare is delivered. In conservative countries such as Israel, there is a noticeable scarcity of information regarding the clinical competency of physiotherapy students (PTSs) in effectively treating lesbian, gay, bisexual and transgender (LGBT) individuals. The objective of this study was to assess the level of LGBT clinical competence among PTSs in Israel.

**Methods:**

Conducted through an anonymous online self-report survey, this study gathered personal and academic background information and self-reported data on previous LGBT education during undergraduate studies of PTSs. It utilized the Hebrew version of the Lesbian, Gay, Bisexual, and Transgender Development of Clinical Skills Scale (LGBT-DOCSS) questionnaire. Descriptive statistics were computed for all outcome measures. The internal reliability of the LGBT-DOCSS was assessed. Total scores of the LGBT-DOCSS, along with scores in each of the three subscales (clinical preparedness, knowledge, and attitudes), were compared across different levels of religiosity and gender.

**Results:**

The sample comprised of 251 PTSs, with an average age of 25.57 ± 3.07 years (34.7% men, 65.3% women). All students reported a lack of LGBT community-related courses during their undergraduate studies. The translated Hebrew version demonstrated good internal consistency, with Cronbach’s alpha ranging from 0.65 to 0.83. The LGBT-DOCSS total score was 4.55 ± 0.61 out of 7, indicating a low level of clinical competency. The highest mean score was in the attitudes subscale (6.55 ± 0.87), which was significantly higher than the scores for the knowledge subscale (3.14 ± 1.46) and clinical preparedness subscale (3.36 ± 0.86). Religiousness was significantly associated with clinical preparedness and attitudes. Men exhibited higher self-reported levels of knowledge and clinical preparedness, albeit with more negative attitudes compare to women. Sexual orientation was significantly associated with clinical competency, with PTSs who identified as heterosexual demonstrating a lower level of clinical competency compared to participants who identified as non-heterosexual.

**Conclusions:**

In Israel, PTSs demonstrated a low level of clinical competency in terms of self-reported knowledge and self-reported clinical preparedness but contrasting positive attitudes toward the LGBT community. Religiousness, gender and sexual orientation had a significant influence on competency levels.These preliminary findings highlight the urgent necessity to enhance the knowledge of PTSs regarding the LGBT community to improve their clinical competence.

**Trial registration NR:**

Not applicable.

## Background

Clinical competence is defined as the knowledge, skills, and abilities needed to deliver safe and effective healthcare services [1]. Appropriate clinical competence requires an understanding of the cultural context in which healthcare is delivered. Healthcare professionals should integrate cultural knowledge, awareness, and skills into their clinical practice, respecting the diverse needs and values of their patients [2, 3]. Clinical competence and cultural competence are closely related concepts that overlap, without a clear distinction between them in healthcare practice [4]. Cultural competence among healthcare professionals encompasses the ability to comprehend and respect cultural differences, including beliefs, values, behaviors, practices, and patient needs, during therapeutic interactions in diverse and multicultural environments [4, 5]. This competency is crucial for providing patient-centered care, creating a non-discriminatory therapeutic setting, mitigating healthcare disparities, and enhancing health outcomes [6, 7]. It is imperative to understand cultural competence as a spectrum, recognizing its broad range and importance in healthcare, rather than perceiving it as a binary concept. Cultural incompetence often leads to the development of an unpleasant therapeutic atmosphere and may result in reluctance in or avoidance seeking medical assistance, thus underutilizing health services. This sequence of events has the potential to harm the physical and mental health of individuals belonging to diverse populations groups [8, 9]. Healthcare organizations worldwide have made efforts to improve cultural competence, with an emphasis on equitable patient-centered care [10, 11]. One of the primary strategies to enhance cultural competence among healthcare providers is through education, specifically by developing cultural competence training programs [12–14].

It is important to consider cultural competency in the context of caring for individuals who identify as lesbian, gay, bisexual, transgender, queer and other sexual and gender diverse communities (LGBTQ+) [15]. However, it has been noted that there is insufficient inclusion of sexual orientation or gender identity in discussions about cultural competence [14, 16]. Neglecting the unique needs and cultural perspectives of LGBTQ + patients contributes to healthcare disparities, including experiences of discrimination and prejudice, as well as the absence of adequate healthcare services specifically tailored to the LGBTQ + community [13, 14]. Given the significant impact of LGBTQ + health and mental health disparities [17], it is imperative to promote and enhance relevant clinical competence.

It has been demonstrated that there are significant differences in LGBT cultural competency levels across healthcare discipline students [18]. Additionally, physiotherapy students (PTSs), along with occupational therapy, pharmacy, and physician assistant students, demonstrated significantly lower LGBT cultural competency than medical and social work students [18]. This difference can be attributed to varying levels of experience with LGBT patients and differences in LGBT curricular education across these disciplines [18].

Previous studies have highlighted a significant lack of attention given to LGBT healthcare issues in the professional curriculum of physical therapists (PTs) and the limited exposure of PTSs to the LGBT community in Australia, the UK and Israel [13, 19, 20]. Previous studies show that PTSs demonstrate favorable attitudes toward LGB individuals [13, 19, 21]. Those attitudes are not necessarily indicative of appropriate preparedness to work with sexual and gender diverse populations, particularly transgender individuals. Cultural competency, in general, encompasses attitudes, skills, and knowledge toward ethnically and racially diverse client groups. It is based on the tripartite model, which combines attitudinal awareness, skills, and knowledge [22].

Two previous studies, a 2016 position paper by Copti et al. [14] in the United States and a 2022 study by Primeau et al. [23] in Canada, emphasized the urgent need for improving cultural competency in physical therapist education [14, 23]. However, there is a dearth of information regarding the clinical competency of PTSs to effectively treat individuals belonging to the LGBT community, particularly in conservative countries such as Israel. This information is crucial for informing PT educational programs and implementing standardized cultural and clinical competency trainings that can potentially enhance routine PT provider behavior, reduce barriers to care, and address health disparities.

Therefore, the primary objective of this study was to explore the clinical competence among PTSs in Israel regarding the LGBT community. This is particularly important due to Israel’s status as a modern, democratic, multicultural state that has a growing acceptance of the LGBT community, particularly among the secular population. Thus we witness increased legislation addressing the rights of sexual minorities [21]. However, notable tensions persist between the orthodox religious groups and the secular community, exacerbated by the militarization and masculinization of Israeli society, potentially fostering an environment of heterosexism and homophobia atmosphere [24–26]. Currently, there is no valid and reliable instrument available in Hebrew for assessing LGBT clinical competency. Therefore, it is crucial to translate an existing questionnaire that meets the criteria of validity and reliability. This translation will enable a comparison of LGBT clinical competence within a multidisciplinary and multinational context. As a result, one of the secondary objectives of this research was to translate and validate the Lesbian, Gay, Bisexual, and Transgender Development of Clinical Skills Scale (LGBT-DOCSS) questionnaire into Hebrew. The questionnaire consists of three subscales: clinical preparedness, knowledge, and attitudes, all of which have been established as reliable and valid [27]. The objectives of this study included evaluating LGBT clinical competency among PTSs in Israel, translating the LGBT-DOCSS into Hebrew, and assessing its internal and discriminative validity.

## Methods

### Procedures

An anonymous online survey was disseminated through various channels to ensure broad participation. The survey was distributed via social media platforms, including Facebook physiotherapy professional groups and student media groups. Additionally, it was shared through the newsletter of the Israeli Physiotherapy Society and through designated representatives in each of the five academic institutions offering a bachelor’s degree in PT. The survey was available between November 2020 and March 2021, with participants accessing it through an anonymous Qualtrics™ link. This study is part of a larger survey, some of which has already been published [21]. Participants were exclusively PTSs in Israel, with no restrictions regarding the number of clinicals or the school year.

### Outcome measure

The data analysis focused on specific sections of the survey, including the following:

1. Background information: Age, sex, gender, sexual orientation, family status, place of residence and religiousness. Participants were asked to identify their level of religiousness, choosing from one of four categories: Very Religious, Religious, Traditional, or Secular. These categories reflect the degree of adherence to traditional religious laws and lifestyles [28].
2. Academic background: Study year, clinical practice experience (yes/no), and the number of rotations in clinical practice.
3. Personal or professional acquaintance with sexual and gender diverse populations was assessed through three yes/no questions: Do you have an LGBTQ + person in your family? Do you have a close friend who identifies as LGBTQ+: Did you treat an LGBTQ + person during your clinical rotation?
4. Assessment of LGBTQ + education during the undergraduate PT program involved two yes/no questions: Did you receive any LGBTQ + community-related courses during your undergraduate studies? Were you exposed to information about the LGBTQ + community as part of your undergraduate studies?
5. Hebrew version of the LGBT-DOCSS - The LGBT-DOCSS was translated into Hebrew with the author’s permission. The translation was completed using the forward and backward translation procedures following recommended guidelines [29]. Forward translation into Hebrew was independently performed by two bilingual physical therapists familiar with the terminology, and they reached a consensus on a single version. Backward translation into Hebrew was conducted by two independent bilingual translators who were not involved in the previous stages, and consensus was reached regarding the accuracy of the version translated back into English. The only modification that was made to the original questionnaire was a glossary that was added at the end of the instructions to familiarize participants with terms (e.g., cisgender: a person whose gender identity and biological sex are compatible).

The LGBT-DOCSS was designed as a self-assessment tool to measure healthcare providers’ clinical competence specific to patients who identify as LGBT. It consists of 18 statements, all of which are ranked on 7-point Likert scales (1 = strongly disagree, 4 = somewhat agree/disagree, 7 = strongly agree). Among the 18 items, eight are reverse scored. The scale comprises three subscales: clinical preparedness (such as “I have received adequate clinical training and supervision to work with transgender clients/patients”), knowledge (such as “I am aware of research indicating that LGB individuals are more likely to be diagnosed with mental illnesses than are heterosexual individuals”), and attitudes (all reverse scored, such as “the lifestyle of an LGB individual is unnatural or immoral”). Statements related to knowledge and preparedness for working with LGB populations and transgender populations appear separately. The instrument has been validated in a large multinational and interdisciplinary sample, demonstrating strong test-retest reliability (0.87) and good internal consistency (all alpha coefficients > 0.80) across its three subscales [27]. To calculate the total score, the averages of all items are taken (referred to as the total LGBT-DOCSS score). Additionally, individual subscale scores are calculated by averaging select items: “clinical preparedness” subscale: seven items, “attitudes” subscale: seven items (reverse scored), “knowledge” subscale: four items. Higher scores on a subscale indicate higher levels of clinical preparedness and knowledge, as well as more positive attitudes toward LGBT patients [27]. Based on a previous study since the original analysis did not specify cutoffs for competency levels, scores of 6 or more (highest 1-point stratification) were considered “high” competency, scores between 5 and 6 were considered “moderate” competency, and scores lower than 5 were considered “low” competency [30].

## Data analysis

Descriptive statistics were calculated for all outcome measures. To assess internal consistency, Cronbach’s alpha coefficient was employed for both the total score of the LGBT-DOCSS and its three subscale scores. A value of α ≥ 0.9 was considered excellent, 0.7 ≤ α < 0.9 was considered good, 0.6 ≤ α < 0.7 was considered acceptable, 0.5 ≤ α < 0.6 was considered poor and values of α < 0.5 were considered unacceptable [31]. The scoring of LGBT-DOCSS questions was carried out in alignment with guidelines provided by Bidell [27]. A comparative analysis was conducted using the independent samples T test to examine mean differences in both the total score and each LGBT-DOCSS subscale across gender and sexual orientation.

One-way ANOVA was employed to explore mean differences for the total score and each LGBT-DOCSS subscale across varying levels of religiosity. If a significant difference was detected, a Scheffe post hoc test was conducted. Significance was set at *p* values ≤ 0.05. The analysis was performed using SAS version 9.4 software.

## Results

The system’s records indicate that there were 320 logins, with 251 respondents successfully completing the questionnaire. Consequently, the sample comprised 251 PTSs from all five physiotherapy education institutes throughout Israel. The sample size fulfilled the criteria outlined in the Consensus-based Standards for the Selection of Health Measurement Instruments (COSMIN) guidelines, which recommend that research focusing on the creation of patient-reported outcome measures should involve a minimum of 100 individuals [32]. On average, it took about 15 min to complete the questionnaire.

The mean age of the participants was 25.57 ± 3.07 years, with 34.7% men and 65.3% women. The background characteristics of the students are detailed in Table 1.

**Table 1:** Participants’ personal and professional background characteristics (*N* = 251)

Age, years mean ± SD, range	25.56 ± 3.07, 19–39
**Sex**, *N* (%)	
male female	87 (34.7) 164 (65.3)
**Gender**, *N* (%)	
Men Women Genderqueer Other	87(34.7) 164(65.3) 0(0) 0(0)
**Sexual orientation**, *N* (%)	
Heterosexual Gay Lesbian Bisexual Queer Other	211(84.1) 9(3.5) 19(7.6) 7(2.8) 0(0) 5(2)
**Religiousness** N (%)	
Very religious Religious Traditional Secular	9(3.6) 27(10.8) 30(12.0) 185(73.7)
**Family status** *N* (%)	
Single Married In a relationship Divorced	125(49.8) 29(11.6) 96(38.2) 10(0.4)
**Place of residence** n (%)	
Urban Other forms of residence (village, kibbutz, etc.)	196 (78.1) 55 (21.9)
Study Year*	
1 2 3 4	105(42.0) 44(17.6) 63(25.2) 38(15.2)
Clinical experience*, *N* (%)	
Yes No	69 (27.5) 182 (72.5)
**Pervious education on LGBTQ + issues**, *N* (%)	
Yes No	0(0) 251 (100.0)
**LGBTQ + family member N (%)**	
Yes No	74 (29.5) 177 (70.5)
**LGBTQ + close friend**, *N* (%)	
Yes No	169 (67.3) 82 (32.7)

* In Israel, five academic institutions offer a bachelor’s degree in PT. All institutions offer full-time four-year programs that include clinicals of at least 960 h during 4–5 rotations

The sample included students from all levels of training, i.e., years one through four, with the majority of the students being 1st-year students (41.8%). Only 69 students (27.5%) had clinical practice experience. Among students with clinical experience, the majority (56 students, 81.16%) reported that they had not had an encounter with a patient who identified as LGBTQ+.

Sexual orientation - The majority of the PTSs (84.1%) identified as heterosexual, while the remaining participants (15.9%) identified as non-heterosexual, including lesbian, gay, bisexual, and other identities. For details, see Table 1.

Personal acquaintance - A total of 29.5% of students had an LGBTQ + person in their family, and 67.3% reported that they had a close friend who identified as LGBTQ+.

LGBTQ + education - All students (100%) reported that they had not received any LGBTQ + community-related courses during their undergraduate studies in physical therapy. However, 16 (6.4%) reported that information about the LGBTQ + community was mentioned as part of their mandatory undergraduate sociology class. Further details on background characteristics are presented in Table 1.

### LGBT clinical competency

The internal consistency coefficient of the translated Hebrew version of the scale was found to be good (total LGBT-DOCSS: Cronbach’s alpha = 0.73, clinical preparedness: 0.65, attitudes: 0.73, knowledge: 0.83).

The mean LGBT-DOCSS total score was 4.55, with a standard deviation of 0.61, indicating a low level of competency. Notably, the highest mean score was in the attitudes subscale (6.55 ± 0.87), which was significantly higher than the scores for the knowledge subscale (3.14 ± 1.46) and clinical preparedness subscale (3.36 ± 0.86). For detailed information, please refer to Table 2. These results indicate that although the PT students demonstrated positive attitudes toward the LGBT community, they showed a contrasting low level of competency in terms of the knowledge and clinical preparedness subscales.

**Table 2:** The results of the lesbian, gay, bisexual, and transgender development of clinical skills scale (LGBT-DOCSS) (mean, standard deviation; *N* = 251)

Clinical preparedness	Mean ± SD	Attitudes subscale	Mean ± SD	Knowledge subscale	Mean ± SD
I would feel unprepared talking with an LGBT client/patient about issues related to their sexual orientation and/or gender identity.^a^	4.78 ± 1.87	I think being transgender is a mental disorder.^a^	6.22 ± 1.44	I am aware of institutional barriers that may inhibit transgender people from using healthcare services.	3.34 ± 2.04
I have received adequate clinical training and supervision to work with transgender clients/patients.	1.65 ± 1.28	A same sex relationship between 2 men or 2 women is not as strong and committed as one between a man and a woman. ^a^	6.70 ± 0.96	I am aware of institutional barriers that may inhibit LGB people from using healthcare services.	2.76 ± 1.75
I have received adequate clinical training and supervision to work with lesbian, gay and bisexual (LGB) clients/patients.	1.90 ± 153	LGB individuals must be discreet about their sexual orientation around children. ^a^	6.43 ± 1.26	I am aware of research indicating that LGB individuals experience disproportionate levels of health and mental health problems compared to heterosexual individuals.	3.18 ± 1.97
I have experience working with LGB clients/patients.	1.88 ± 1.60	In regard to transgender individuals, I believe they are morally deviant. ^a^	6.63 ± 1.04	I am aware of research indicating that transgender individuals experience disproportionate levels of health and mental problems compared to cisgender individuals.	3.28 ± 2.08
I feel competent to assess a person who is LGB in a therapeutic setting.	6.11 ± 1.48	The lifestyle of an LGB individual is unnatural or immoral. ^a^	6.61 ± 1.15		
I feel competent to assess a person who is transgender in a therapeutic setting.	5.80 ± 1.63	People who dress opposite to their biological sex have a perversion ^a^	6.49 ± 1.18		
I have experience working with transgender clients/patients.	1.37 ± 1.14	I would be morally uncomfortable working with an LGBT client/patient.^a^	6.74 ± 0.92		
**Total**	3.36 ± 0.86		6.55 ± 0.87		3.14 ± 1.46

^a^- reverse scored

The comparison of LGBT-DOCSS scores and its three subscale scores across different levels of religiousness is presented in Table 3. Overall, Clinical competency levels were generally low, with mean scores below 5 across all religiousness levels. Notably, a statistically significant difference in overall LGBT-DOCSS scores was observed among the various levels of religiousness (*p* < 0.0001). Secular individuals demonstrated the highest level of clinical competency, while those identifying as religious had the lowest scores. Regarding the subscales scores, there was a significant difference in clinical preparedness and attitudes across varying levels of religiousness. For clinical preparedness, a statistically significant difference was identified between secular and traditional individuals (*p* = 0.0011), with secular participants reporting higher clinical preparedness levels In the attitudes subscale, individuals who self-identified as religious or very religious had significantly lower scores compared to those who were traditional or secular (*p* < 0.0001). Conversely, no significant differences were observed in the knowledge subscale scores across religiousness levels.

**Table 3:** Comparing the LGBT-DOCSS and subscales across varying religiousness levels

Variable (mean ± standard deviation)	Secular (*N* = 185)	Traditional (*N* = 30)	Very religious and religious (*N* = 36)	F(df), *p* value
**Total score of the LGBT-DOCSS**	4.7 ± 0.5	4.4 ± 0.7	4.0 ± 0.6	F (2.248) = 27.0, *p* < 0.0001
Clinical Preparedness	3.5 ± 0.8	2.9 ± 0.9	3.1 ± 0.8	F (2.248) = 6.98, *p* = 0.0011
Knowledge	3.2 ± 1.5	3.1 ± 1.5	3.1 ± 1.3	F (2.248) = 0.08, *p* = 0.92
Attitudes	6.8 ± 0.5	6.5 ± 0.7	5.3 ± 1.4	F (2.248) = 61.0, *p* < 0.0001

LGBT-DOCSS: Lesbian, Gay, Bisexual, and Transgender Development of Clinical Skills Scale, ±standard deviation

A comparison of the values of the LGBT-DOCSS and its three subscales between genders) mean and standard deviation) is presented in Table 4. When analyzing gender differences, no significant difference was observed in the total scores of the LGBT-DOCSS. A statistically significant difference was observed between the genders in all three subscales, with men demonstrating higher self-reported levels of knowledge and self-reported clinical preparedness but also displaying more negative attitudes compared to women.

**Table 4:** Comparing the LGBT-DOCSS and subscales between genders

Variable (mean ± standard deviation)	Men (*N* = 87)	Women (*N* = 164)	T (df), *p* value
**Total score of the LGBT-DOCSS**	4.6 ± 0.6	4.5 ± 0.6	T (249) = 0.84, *p* = 0.4
Clinical Preparedness	3.5 ± 0. 7	3.3 ± 0.9	T (210.19) = 2.39, *p* = 0.02
Knowledge	3.4 ± 1.4	3.0 ± 1.5	T (249) = 2.06, *p* = 0.04
Attitudes	6.4 ± 1.1	6.7 ± 0.7	T (127.31) =-2.34, *p* = 0.02

Table 5 presents a comparison of the mean and standard deviation values of the LGBT-DOCSS and its three subscales between heterosexual and non-heterosexual participants.

A statistically significant difference was observed in the total scores of the LGBT-DOCSS and all three subscales, with non-heterosexual participants demonstrating a higher level of clinical competence. This was reflected in their self-reported higher levels of clinical preparedness and knowledge, as well as more positive attitudes toward the LGBT community.

**Table 5:** Comparing the LGBT-DOCSS and subscales between heterosexuals and not heterosexuals

Variable (mean±:standard deviation)	Heterosexual (*N* = 211)	Not heterosexuals (*N* = 40)	T(df), *p* value
Total score of the LGBT-DOCSS	4.5 ± 0.6	4.9 ± 0.5	T (249) = 3.88, *p* < 0.001
Clinical Preparedness	3.3 ± 0.9	3.7 ± 0.7	T (249) = 2.46, *p* = 0.01
Knowledge	3.0 ± 1.4	3.7 ± 1.6	T (249) = 2.68, *p* = 0.008
Attitudes	6.5 ± 0.9	6.8 ± 0.4	T (136.13) =-3.22, *p* = 0.002

## Discussion

### LGBT clinical competency among physiotherapy students

This study is the first to assess the competency of physiotherapy students in Israel regarding LGBT patients. These preliminary results illustrate that PTSs exhibited a low level of clinical competency, as indicated by a total LGBT-DOCSS score of 4.55 (± 0.61) out of 7. Two previous studies [18, 33] have delved into clinical competency regarding working with LGBT individuals among PTSs utilizing the same LGBT-DOCSS tool employed in this study. Nowaskie et al. [18] assessed clinical competence related to LGBT issues among students in various healthcare disciplines in the USA. Their study included a sample of 1701 healthcare professional students, 42 (2.5%) of whom were physical therapists. The findings closely parallel the results of our study, demonstrating that students exhibited a low level of preparedness, with LGBT-DOCSS scores averaging 4.96 (± 0.8). Primeau et al. [33] showed a moderate level of competency among 15 PTSs in Canada, as evidenced by a total LGBT-DOCSS score of 5.10 (± 0.66) out of 7. It is possible that the slight increase in competency level (a score difference of 0.55) compared to the current study might be attributed to differences in characteristics, notably gender and self-identified sexual orientation. These are recognized as factors that can influence competency level [33, 34].

Low clinical competency has been linked to the emergence of health disparities in healthcare settings, including issues such as discrimination and barriers to accessing by the LGBTI community [35, 36] Ross and Setchell [37] demonstrated that LGBTQ patients may experience discomfort, discrimination, due to a lack of relevant knowledge when treated by physiotherapists. They also highlighted that physiotherapist may create negative encounters and biases within the healthcare setting.

### LGBT clinical competency across different levels of religiosity

The current study demonstrated significant differences in clinical competency among various levels of religiousness. The highest level of clinical competency was observed among secular individuals, while the lowest was found among those who identified as religious. An analysis of the subscales within the total LGBT-DOCSS score revealed that the lower level of cultural competence among elderly people was primarily attributed to more negative attitudes toward LGBT individuals compared to their secular counterparts. Additionally, there was a lower level of clinical readiness. However, it is worth noting that a direct comparison of these findings was impossible due to the absence of similar studies that have examined differences in LGBT-DOCSS scores in relation to the subjects’ religious affiliations. Yet, the presence of more negative attitudes toward LGBT individuals among religious PTSs and registered PTs compared to secular individuals has been previously documented in Israel [15, 21]. Janssen and Scheepers [38] found that globally, individuals who consider their religion to be the only accepted religion are deeply integrated within their religious community and adhere to traditional gender roles and tend to hold more negative views toward homosexuality than others. Furthermore, there is a large body of evidence demonstrating a connection between religious affiliation and negative attitudes toward LGBT individuals among healthcare professionals, which also extends to increased discomfort when examining and treating patients who identify as part of the LGBT community [9, 20, 33, 34, 39–41].

A possible explanation for the significantly lower level of self-reported clinical preparedness observed in the current study among religious students, in comparison to their secular counterparts as revealed in this study, may be rooted in the students’ choices for reduced exposure to this population during their studies. This is because exposure has been identified as one of the factors that influences the level of clinical preparedness [23]. This hypothesis could not be evaluated within the confines of the available data, as there were no data regarding the students’ exposure to LGBT patients from the community regarding the level of religiosity. Further research is warranted to explore the potential association between religiosity and the knowledge, self-reported clinical preparedness, and actual clinical preparedness of pre-clinical medical students. This might entail adjusting curricular components and potentially creating a specialized subspecialty for students interested in serving specific communities, with considerations regarding their religious affiliation.

### LGBT clinical competency across genders

The current study’s results indicate a significant influence of gender on all three subscales of clinical competency among PTSs. Specifically, men demonstrated greater self-reported levels of knowledge and clinical preparedness, although they displayed more negative attitudes compared to their female counterparts. However, there was no discernible significant difference between the genders in terms of the total LGBT-DOCSS score. This finding warrants further exploration. The current finding that men have more negative attitudes than women was also found in a previous study that examined the attitudes of PTSs in Israel [21], as well as among students of other health professions who also used the tool and tested the effect of gender on clinical competency [42, 43]. The results of the current study revealed that men demonstrated higher levels of self-reported clinical preparedness than women. This finding aligns with the results reported by a prior study by Nowaskie and Najam [42] involving health profession students, medical students specializing in psychiatry and dementia caregivers. Nowaskie and Najam [42] explained that, as the LGBT-DOCSS questionnaire relies on self-reports from the subjects regarding their level of readiness, women’s self-assessment might be influenced by unconscious biases regarding their abilities. In contrast, Badat et al. [43] found that the gender of medical students had no influence on the scores of the clinical preparedness subscale in the LGBT-DOCSS questionnaire. Regarding the subscale of knowledge in the LGBT-DOCSS, this study found that men possessed more knowledge than women. However, the two previous studies cited above [42, 43], which focused on health professionals other than physical therapists, reported contrasting results, where women exhibited higher knowledge levels than men. Consequently, it appears that there is a need for further research into the role of gender in healthcare provision to LGBT patients by PTSs.

### LGBT clinical competency based on sexual orientation

The current results indicate that PTSs who identify as non-heterosexual exhibit a statistically significant higher level of clinical competence compared to their heterosexual counterparts. This finding aligns with the previous study of Nowaskie et al. [42]. , which explored LGBT cultural competency among 2254 healthcare professionals using the same tool (LGBT-DOCSS) employed in our study. They demonstrated that sexual minority professionals scored higher on the LGBT-DOCSS, reflecting greater LGBT competence compared to heterosexual professionals. These results were attributed to the personal identification, values, and experiences of sexual minority professionals with stigma and discrimination. Consequently, these professionals may develop enhanced competence through increased recognition and awareness of LGBT healthcare, personal experiences with multiple minority identities, and the pursuit of advanced education and training. To date, only one study [18] has examined the LGBT clinical competence of PTSs, along with students from other healthcare disciplines such as occupational therapy, pharmacy, and physician assistant programs. This study included a total sample of 1701 students from three universities across the United States and utilized the LGBT-DOCSS tool to assess clinical competency. It is noteworthy that physical therapy students comprised a small percentage of the total sample (2.5%, 42 participants). Among these physical therapy students, 88.1% identified as heterosexual, a percentage comparable to the 84.1% found in our current study. However, this study did not analyze the impact of sexual orientation on clinical competence. Accordingly, it is not possible to compare the research findings to previous studies, and this indicates the need for follow-up studies to strengthen the findings of the current study.

### Self-reported knowledge of physiotherapy students about the LGBT community

The present study found that all students (100%) reported not having received any LGBTQ + community-related courses during their undergraduate studies in physical therapy. This concerning situation aligns with findings from previous studies among PTs and PTSs in Israel [15, 21] and underscores the position paper by Copti et al. [14] in 2016 and Primeau et al. [23] in 2022 emphasizing the urgent need to enhance the knowledge of PTs regarding the LGBTQ + community to enhance clinical competence. This enhancement is based on the tripartite model of clinical competence, which encompasses the Attitudes subscale, skills, and knowledge [22].

### Hebrew version of the LGBT-DOCSS questionnaire

A secondary objective of this study was the translation and validation of the LGBT-DOCSS questionnaire into Hebrew. The research demonstrated that the internal consistency coefficient of the overall score was good, with a Cronbach’s alpha of 0.73, which was slightly lower than that of the original English version (Cronbach’s alpha = 0.86) [31]. The values for the subscales were as follows: the knowledge subscale value (Cronbach’s α = 0.83) was consistent with that of the original version (Cronbach’s α = 0.83) [31]. The remaining two subscales had slightly lower values compared to the original version (clinical preparedness: 0.65 versus a value of 0.88 in the original version, and attitudes subscale: 0.73 versus a value of 0.8 in the original version). Nevertheless, the current Cronbach’s alpha coefficients confirm that the Hebrew version maintains good internal reliability. Therefore, it can be considered a reliable tool for evaluating clinical competency among health professionals in Israel.

### Study limitations and further research

Several study limitations should be considered. We utilized a tool that assesses explicit attitudes and self-reported clinical preparedness, and it is possible that examining the implicit attitudes and actual clinical preparedness of PTSs could yield different results. Regarding our recruitment method, which involved using social media and a snowballing approach, there were several drawbacks: compliance percentages could not be calculated as the questionnaires were distributed online, and we could not determine the total number of PTSs who received the link but chose not to complete the questionnaires. In addition, this approach might have implications for the representativeness of the population of interest. Specifically, individuals with strong sexual prejudices or biases, whether positive or negative, toward sexual and gender diverse populations may have been more likely to complete the survey, while those with more moderate views might have opted not to participate, as no rewards were offered to incentivize their involvement. Future research should account for participants’ sexual orientation and gender identities, which may have influenced the outcomes. Additionally, subsequent studies should assess LGBT clinical competency of PTSs through practical evaluations, including objective tests of knowledge and clinical skills.

## Conclusions

The preliminary results revealed that PTSs in Israel exhibit a low level of competency regarding working with LGBT individuals. This study highlights a gap among overall positive attitudes, low clinical preparedness, and even lower self-reported levels of knowledge. Religiousness, gender and sexual orientation had a significant influence on competency levels. These findings highlight the urgent necessity to enhance the knowledge of PTSs’ regarding the LGBT community in order to improve their clinical competence.

## Data Availability

Data are available upon reasonable request from the corresponding author (Michal Elboim-Gabyzon; michal.elboim@gmail.com, moelboum-@staff.haifa.ac.il.

## Abbreviations

PT: physiotherapy
PTs: physical therapists
PTSs: physiotherapy students
LGBT: lesbian, gay, bisexual and transgender
LGBTQ: lesbian, gay, bisexual and transgender and queer/ questioning
LGBTQ+: lesbian, gay, bisexual, transgender, queer/questioning and other sexual and gender diverse populations
LGBT-DOCSS: Lesbian, Gay, Bisexual, and Transgender Development of Clinical Skills Scale

## References

[1] Peterson S (2022). The blind men, the elephant, and the continuing education course: why higher standards are needed in physical therapist professional development. J Orthop Sports Phys Therapy.

[2] Dickson T (2022). Transforming the patient experience: moving beyond cultural competence to cultural safety. Phys Ther.

[3] Bolding DJ (2020). Survey of occupational therapy students’ attitudes, knowledge and preparedness for treating LGBT clients. J Occup Therapy Educ.

[4] Yamada A-M, Brekke JS (2008). Addressing mental health disparities through clinical competence not just cultural competence: the need for assessment of sociocultural issues in the delivery of evidence-based psychosocial rehabilitation services. Clin Psychol Rev.

[5] Nair L, Adetayo OA. Cultural competence and ethnic diversity in healthcare. Plast Reconstr Surg Global Open, 2019. 7(5). 10.1097/GOX.0000000000002219 PMC6571328 31333951

[6] Miller KL (2016). Patient centered care: a path to better health outcomes through engagement and activation. NeuroRehabilitation.

[7] Hayward LM, Li L (2014). Promoting and assessing cultural competence, professional identity, and advocacy in doctor of physical therapy (DPT) degree students within a community of practice. J Phys Therapy Educ.

[8] Watters A, Bergstrom A, Sandefer R. PATIENT ENGAGEMENT AND MEANINGFUL USE: ASSESSING THE IMPACT OF THE EHR INCENTIVE PROGRAM ON CULTURAL COMPETENCE IN HEALTHCARE. J Cult Divers, 2016. 23(3). 29694753

[9] Watson H (2019). A systematic review of ethnic minority women’s experiences of perinatal mental health conditions and services in Europe. PLoS ONE.

[10] Gupta AD. *A Systematic Review of the Literature on the Development of New Concepts from the Perspective of Promoting Patient-Centered Care* 2023.

[11] Kwame A, Petrucka PM (2021). A literature-based study of patient-centered care and communication in nurse-patient interactions: barriers, facilitators, and the way forward. BMC Nurs.

[12] Ozcan Edeer A, Rust N (2022). Effectiveness of interprofessional education modules on cultural competency of physical therapy and occupational therapy students. Internet J Allied Health Sci Pract.

[13] Morton RC (2021). Addressing Lesbian, Gay, Bisexual, Transgender, and Queer Health in Physical Therapy Education. J Phys Therapy Educ.

[14] Copti N (2016). Lesbian, gay, bisexual, and transgender inclusion in physical therapy: advocating for cultural competency in physical therapist education across the United States. J Phys Therapy Educ.

[15] Klein R, Elboim-Gabyzon M (2021). Attitudes of registered physiotherapists in Israel toward people identifying as lesbian, gay, and bisexual: a cross-sectional survey. BMC Med Educ.

[16] Butler M et al. *Improving cultural competence to reduce health disparities* 2016. 27148614

[17] Medina-Martínez J (2021). Health inequities in LGBT people and nursing interventions to reduce them: a systematic review. Int J Environ Res Public Health.

[18] Nowaskie DZ, Patel AU, Fang RC (2020). A multicenter, multidisciplinary evaluation of 1701 healthcare professional students’ LGBT cultural competency: comparisons between dental, medical, occupational therapy, pharmacy, physical therapy, physician assistant, and social work students. PLoS ONE.

[19] Brenner N et al. Physiotherapy students’ education on, exposure to, and attitudes and beliefs about providing care for LGBTQIA + patients: a cross-sectional study in the UK. Eur J Physiotherapy, 2022: p. 1–9.

[20] Ross M, Setchell J (2021). Physiotherapists’ knowledge regarding and experience working with clients who identify as LGBTQIA+. J Sci Med Sport.

[21] Elboim-Gabyzon M, Ofek H, Klein R. *Attitudes of Physical Therapy Students in Israel toward People Identifying as Lesbian, Gay, or Bisexual: A Cross-Sectional Survey* Health & Social Care in the Community, 2023. 2023.

[22] Pynor R, Weerakoon P, Jones MK (2005). A preliminary investigation of physiotherapy students’ attitudes towards issues of sexuality in clinical practice. Physiotherapy.

[23] Primeau CA, et al. A need for Greater emphasis on 2SLGBTQIA + health among physiotherapists in Canada. University of Toronto; 2022. pp. 117–20. 10.3138/ptc-2021-0107-gee PMC10262744 37323717

[24] Davidovitz M. *Winds of change: How street-level bureaucrats actively represent minority clients by influencing majority clients—The context of LGB Israeli teachers* Public Administration, 2022.

[25] Marciano A, Antebi-Gruszka N (2022). Offline and online discrimination and mental distress among lesbian, gay, and bisexual individuals: the moderating effect of LGBTQ facebook use. Media Psychol.

[26] Shilo G. *Attitudes toward homosexuality among social work students* Unpublished master’s thesis. Tel Aviv University, Tel Aviv.(Hebrew), 2004.

[27] Bidell MP, Stepleman LM (2017). An interdisciplinary approach to lesbian, gay, bisexual, and transgender clinical competence, professional training, and ethical care: introduction to the special issue. J Homosex.

[28] Sharabi M, Kay A. *Work Values of Working Women in Israel: A Comparison of Haredi Women with Those from the Secular and Traditional Segments* Contemporary Jewry, 2023: pp. 1–21. 10.1007/s12397-023-09480-3 PMC10116109 37360648

[29] Guillemin F, Bombardier C, Beaton D (1993). Cross-cultural adaptation of health-related quality of life measures: literature review and proposed guidelines. J Clin Epidemiol.

[30] Nowaskie DZ, Patel AU (2020). How much is needed? Patient exposure and curricular education on medical students’ LGBT cultural competency. BMC Med Educ.

[31] Bland JM, Altman DG (1997). Statistics notes: Cronbach’s alpha. BMJ.

[32] Mokkink LB (2016). The COnsensus-based standards for the selection of health measurement INstruments (COSMIN) and how to select an outcome measurement instrument. Braz J Phys Ther.

[33] Primeau CA (2023). Students’ attitudes, beliefs and perceptions surrounding 2SLGBTQIA + health education and inclusiveness in Canadian physiotherapy programs. BMC Public Health.

[34] Fisher AD (2017). Who has the worst attitudes toward sexual minorities? Comparison of transphobia and homophobia levels in gender dysphoric individuals, the general population and health care providers. J Endocrinol Investig.

[35] Zeeman L (2019). A review of lesbian, gay, bisexual, trans and intersex (LGBTI) health and healthcare inequalities. Eur J Pub Health.

[36] Burch A (2008). Health care providers’ knowledge, attitudes, and self-efficacy for working with patients with spinal cord injury who have diverse sexual orientations. Phys Ther.

[37] Ross MH, Setchell J (2019). People who identify as LGBTIQ + can experience assumptions, discomfort, some discrimination, and a lack of knowledge while attending physiotherapy: a survey. J Physiotherapy.

[38] Janssen D-J, Scheepers P. How religiosity shapes rejection of homosexuality across the globe. J Homosex, 2018. 10.1080/00918369.2018.1522809 30372378

[39] Ensign KA (2011). Athletic trainers’ attitudes toward lesbian, gay, and bisexual National Collegiate Athletic Association student-athletes. J Athl Train.

[40] Jäckle S, Wenzelburger G (2015). Religion, religiosity, and the attitudes toward homosexuality—A multilevel analysis of 79 countries. J Homosex.

[41] Nye EA (2019). Lesbian, gay, bisexual, transgender, and queer patients: collegiate athletic trainers’ perceptions. J Athl Train.

[42] Nowaskie DZ, Najam S (2022). Lesbian, gay, bisexual, and/or transgender (LGBT) cultural competency across the intersectionalities of gender identity, sexual orientation, and race among healthcare professionals. PLoS ONE.

[43] Badat A, Moodley S, Paruk L (2023). Preparedness of final year medical students in caring for lesbian, gay, bisexual, and transgender patients with mental illness. South Afr J Psychiatry.

